# A practical guide to single-cell RNA-sequencing for biomedical research and clinical applications

**DOI:** 10.1186/s13073-017-0467-4

**Published:** 2017-08-18

**Authors:** Ashraful Haque, Jessica Engel, Sarah A. Teichmann, Tapio Lönnberg

**Affiliations:** 1 QIMR Berghofer Medical Research Institute, Herston, Brisbane, Queensland 4006 Australia; 2 Wellcome Trust Sanger Institute, Wellcome Genome Campus, Hinxton, Cambridge, CB10 1SA UK; 3 Turku Centre for Biotechnology, University of Turku and Åbo Akademi University, FI-20520 Turku, Finland

## Abstract

RNA sequencing (RNA-seq) is a genomic approach for the detection and quantitative analysis of messenger RNA molecules in a biological sample and is useful for studying cellular responses. RNA-seq has fueled much discovery and innovation in medicine over recent years. For practical reasons, the technique is usually conducted on samples comprising thousands to millions of cells. However, this has hindered direct assessment of the fundamental unit of biology—the cell. Since the first single-cell RNA-sequencing (scRNA-seq) study was published in 2009, many more have been conducted, mostly by specialist laboratories with unique skills in wet-lab single-cell genomics, bioinformatics, and computation. However, with the increasing commercial availability of scRNA-seq platforms, and the rapid ongoing maturation of bioinformatics approaches, a point has been reached where any biomedical researcher or clinician can use scRNA-seq to make exciting discoveries. In this review, we present a practical guide to help researchers design their first scRNA-seq studies, including introductory information on experimental hardware, protocol choice, quality control, data analysis and biological interpretation.

## Background

Medicine now exists in a cellular and molecular era, where experimental biologists and clinicians seek to understand and modify cell behaviour through targeted molecular approaches. To generate a molecular understanding of cells, the cells can be assessed in a variety of ways, for example through analyses of genomic DNA sequences, chromatin structure, messenger RNA (mRNA) sequences, non-protein-coding RNA, protein expression, protein modifications and metabolites. Given that the absolute quantity of any of these molecules is very small in a single living cell, for practical reasons many of these molecules have been assessed in ensembles of thousands to billions of cells. This approach has yielded much useful molecular information, for example in genome-wide association studies (GWASs), where genomic DNA assessments have identified single-nucleotide polymorphisms (SNPs) in the genomes of individual humans that have been associated with particular biological traits and disease susceptibilities.

To understand cellular responses, assessments of gene expression or protein expression are needed. For protein expression studies, the application of multi-colour flow cytometry and fluorescently conjugated monoclonal antibodies has made the simultaneous assessment of small numbers of proteins on vast numbers of single cells commonplace in experimental and clinical research. More recently, mass cytometry (Box 1), which involves cell staining with antibodies labelled with heavy metal ions and quantitative measurements using time-of-flight detectors, has increased the number of proteins that can be assessed by five- to tenfold [1, 2] and has started to reveal previously unappreciated levels of heterogeneity and complexity among apparently homogeneous cell populations, for example among immune cells [1, 3]. However, it remains challenging to examine simultaneously the entire complement of the thousands of proteins (known as the ‘proteome’) expressed by the genome that exist in a single cell.

As a proxy for studying the proteome, many researchers have turned to protein-encoding, mRNA molecules (collectively termed the ‘transcriptome’), whose expression correlates well with cellular traits and changes in cellular state. Transcriptomics was initially conducted on ensembles of millions of cells, firstly with hybridization-based microarrays, and later with next-generation sequencing (NGS) techniques referred to as RNA-seq. RNA-seq on pooled cells has yielded a vast amount of information that continues to fuel discovery and innovation in biomedicine. Taking just one clinically relevant example—RNA-seq was recently performed on haematopoietic stem cells to stratify acute myeloid leukaemia patients into cohorts requiring differing treatment regimens [4]. Nevertheless, the averaging that occurs in pooling large numbers of cells does not allow detailed assessment of the fundamental biological unit—the cell—or the individual nuclei that package the genome.

Since the first scRNA-seq study was published in 2009 [5], there has been increasing interest in conducting such studies. Perhaps one of the most compelling reasons for doing so is that scRNA-seq can describe RNA molecules in individual cells with high resolution and on a genomic scale. Although scRNA-seq studies have been conducted mostly by specialist research groups over the past few years [5–16], it has become clear that biomedical researchers and clinicians can make important new discoveries using this powerful approach as the technologies and tools needed for conducting scRNA-seq studies have become more accessible. Here, we provide a practical guide for biomedical researchers and clinicians who might wish to consider performing scRNA-seq studies.

## Box 1. Glossary

**Barcoding** Tagging single cells or sequencing libraries with unique oligonucleotide sequences (that is, ‘barcodes’), allowing sample multiplexing. Sequencing reads corresponding to each sample are subsequently deconvoluted using barcode sequence information.

**Dropout** An event in which a transcript is not detected in the sequencing data owing to a failure to capture or amplify it.

**Mass cytometry** A technique based on flow cytometry and mass spectrometry, in which protein expression is interrogated using antibodies labelled with elemental tags—allows parallel measurements of dozens of proteins on thousands of single cells in one experiment.

**Sequencing depth** A measure of sequencing capacity spent on a single sample, reported for example as the number of raw reads per cell.

**Spike-in** A molecule or a set of molecules introduced to the sample in order to calibrate measurements and account for technical variation; commonly used examples include external RNA control consortium (ERCC) controls (Ambion/Thermo Fisher Scientific) and Spike-in RNA variant control mixes (SIRVs, Lexogen).

**Split-pooling** An approach where sample material is subjected to multiple rounds of aliquoting and pooling, often used for producing unique barcodes by step-wise introduction of distinct barcode elements into each aliquot.

**Transcriptional bursting** A phenomenon, also known as ‘transcriptional pulsing’, of relatively short transcriptionally active periods being followed by longer silent periods, resulting in temporal fluctuation of transcript levels.

**Unique molecular identifier** A variation of barcoding, in which the RNA molecules to be amplified are tagged with random n-mer oligonucleotides. The number of distinct tags is designed to significantly exceed the number of copies of each transcript species to be amplified, resulting in uniquely tagged molecules, and allowing control for amplification biases.

## Why consider performing scRNA-seq?

scRNA-seq permits comparison of the transcriptomes of individual cells. Therefore, a major use of scRNA-seq has been to assess transcriptional similarities and differences within a population of cells, with early reports revealing previously unappreciated levels of heterogeneity, for example in embryonic and immune cells [9, 10, 17]. Thus, heterogeneity analysis remains a core reason for embarking on scRNA-seq studies.

Similarly, assessments of transcriptional differences between individual cells have been used to identify rare cell populations that would otherwise go undetected in analyses of pooled cells [18], for example malignant tumour cells within a tumour mass [19], or hyper-responsive immune cells within a seemingly homogeneous group [13]. scRNA-seq is also ideal for examination of single cells where each one is essentially unique, such as individual T lymphocytes expressing highly diverse T-cell receptors [20], neurons within the brain [15] or cells within an early-stage embryo [21]. scRNA-seq is also increasingly being used to trace lineage and developmental relationships between heterogeneous, yet related, cellular states in scenarios such as embryonal development, cancer, myoblast and lung epithelium differentiation and lymphocyte fate diversification [11, 21–25].

In addition to resolving cellular heterogeneity, scRNA-seq can also provide important information about fundamental characteristics of gene expression. This includes the study of monoallelic gene expression [9, 26, 27], splicing patterns [12], as well as noise during transcriptional responses [7, 12, 13, 28, 29]. Importantly, studying gene co-expression patterns at the single-cell level might allow identification of co-regulated gene modules and even inference of gene-regulatory networks that underlie functional heterogeneity and cell-type specification [30, 31].

Yet, while scRNA-seq can provide answers to many research questions, it is important to understand that the details of any answers provided will vary according to the protocol used. More specifically, the level of detail that can be resolved from the mRNA data, such as how many genes can be detected, and how many transcripts of each gene can be detected, whether a specific gene of interest is expressed, or whether differential splicing has occurred, depends on the protocol. Comparisons between protocols in terms of their sensitivity and specificity have been discussed by Ziegenhain et al. [32] and Svensson et al. [33].

## What are the basic steps in conducting scRNA-seq?

**Fig. 1:**
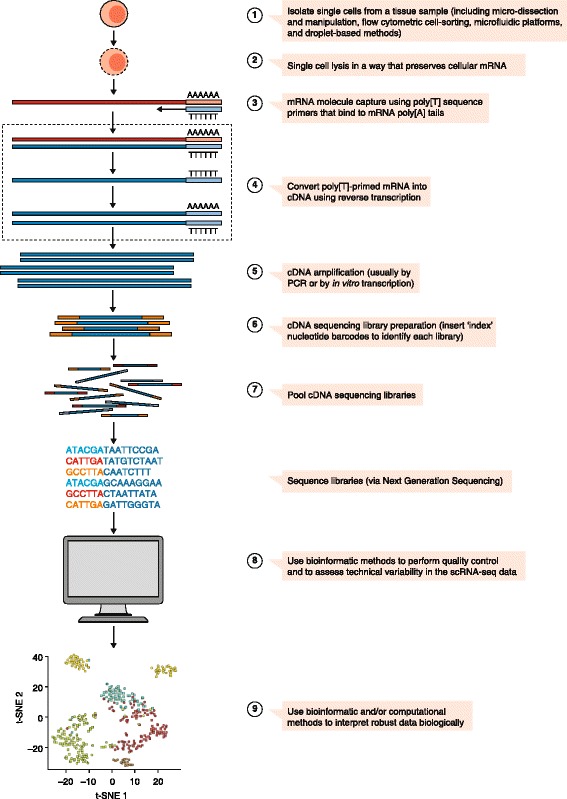
General workflow of single-cell RNA-sequencing (scRNA-seq) experiments. A typical scRNA-seq workflow includes most of the following steps: 1) isolation of single cells, 2) cell lysis while preserving mRNA, 3) mRNA capture, 4) reverse transcription of primed RNA into complementary DNA (cDNA), 5) cDNA amplification, 6) preparation of cDNA sequencing library, 7) pooling of sequence libraries, 8) use of bio-informatic tools to assess quality and variability, and 9) use of specialized tools to analyse and present the data. *t-SNE* t-distributed stochastic neighbour embedding

Although many scRNA-seq studies to date have reported bespoke techniques, such as new developments in wet-lab, bio-informatic or computational tools, most have adhered to a general methodological pipeline (Fig. 1). The first, and most important, step in conducting scRNA-seq has been the effective isolation of viable, single cells from the tissue of interest. We point out here, however, that emerging techniques, such as isolation of single nuclei for RNA-seq [34–36] and ‘split-pooling’ (Box 1) scRNA-seq approaches, based on combinatorial indexing of single cells [37, 38], provide certain benefits over isolation of single intact cells, such as allowing easier analyses of fixed samples and avoiding the need for expensive hardware. Next, isolated individual cells are lysed to allow capture of as many RNA molecules as possible. In order to specifically analyse polyadenylated mRNA molecules, and to avoid capturing ribosomal RNAs, poly[T]-primers are commonly used. Analysis of non-polyadenylated mRNAs is typically more challenging and requires specialized protocols [39, 40]. Next, poly[T]-primed mRNA is converted to complementary DNA (cDNA) by a reverse transcriptase. Depending on the scRNA-seq protocol, the reverse-transcription primers will also have other nucleotide sequences added to them, such as adaptor sequences for detection on NGS platforms, unique molecular identifiers (UMIs; Box 1) to mark unequivocally a single mRNA molecule, as well as sequences to preserve information on cellular origin [41]. The minute amounts of cDNA are then amplified either by PCR or, in some instances, by in vitro transcription followed by another round of reverse transcription—some protocols opt for nucleotide barcode-tagging (Box 1) at this stage to preserve information on cellular origin [42]. Then, amplified and tagged cDNA from every cell is pooled and sequenced by NGS, using library preparation techniques, sequencing platforms and genomic-alignment tools similar to those used for bulk samples [43]. The analysis and interpretation of the data comprise a diverse and rapidly developing field in itself and will be discussed further below.

It is important to note that commercial kits and reagents now exist for all the wet-lab steps of a scRNA-seq protocol, from lysing cells through to preparing samples for sequencing. These include the ‘switching mechanism at 5’ end of RNA template’ (SMARTer) chemistry for mRNA capture, reverse transcription and cDNA amplification (Clontech Laboratories). Furthermore, commercial reagents also exist for preparing barcoded cDNA libraries, for example Illumina’s Nextera kits. Once single cells have been deposited into individual wells of a plate, these protocols, and others from additional commercial suppliers (for example, BD Life Sciences/Cellular Research), can be conducted without the need for further expensive hardware other than accurate multi-channel pipettes, although it should be noted that, in the absence of a microfluidic platform in which to perform scRNA-seq reactions (for example, the C1 platform from Fluidigm), reaction volumes and therefore reagent costs can increase substantially. Moreover, downscaling the reactions to nanoliter volumes has been shown to improve detection sensitivity [33] and quantitative accuracy [44].

More recently, droplet-based platforms (for example, Chromium from 10x Genomics, ddSEQ from Bio-Rad Laboratories, InDrop from 1CellBio, and μEncapsulator from Dolomite Bio/Blacktrace Holdings) have become commercially available, in which some of the companies also provide the reagents for the entire wet-lab scRNA-seq procedure. Droplet-based instruments can encapsulate thousands of single cells in individual partitions, each containing all the necessary reagents for cell lysis, reverse transcription and molecular tagging, thus eliminating the need for single-cell isolation through flow-cytometric sorting or micro-dissection [45–47]. This approach allows many thousands of cells to be assessed by scRNA-seq. However, a dedicated hardware platform is a prerequisite for such droplet-based methods, which might not be readily available to a researcher considering scRNA-seq for the first time. In summary, generating a robust scRNA-seq dataset is now feasible for wet-lab researchers with little to no prior expertise in single-cell genomics. Careful consideration must be paid, however, to the commercial protocols and platforms to be adopted. We will discuss later which protocols are favoured for particular research questions.

## What types of material can be assessed by scRNA-seq?

Many of the initial scRNA-seq studies successfully examined human or mouse primary cells, such as those from embryos [17], tumours [14], the nervous system [15, 48] and haematopoietically derived cells, including stem cells and fully differentiated lymphocytes [8, 16, 49, 50]. These studies suggested that, in theory, any eukaryotic cell can be studied using scRNA-seq. Consistent with this, a consortium of biomedical researchers has recently committed to employ scRNA-seq for creating a transcriptomic atlas of every cell type in the human body—the Human Cell Atlas [51]. This will provide a highly valuable reference for future basic research and translational studies.

Although there is great confidence in the general utility of scRNA-seq, one technical barrier must be carefully considered—the effective isolation of single cells from the tissue of interest. While this has been relatively straightforward for immune cells in peripheral blood or loosely retained in secondary lymphoid tissue, and certainly has been achievable for excised tumours, this could be quite different for many other tissues, in which single cells can be cemented to extracellular-scaffold-like structures and to other neighbouring cells. Although commercial reagents exist for releasing cells from such collagen-based tethers (for example, MACS Tissue Dissociation kits from Miltenyi Biotec), there remains significant theoretical potential for these protocols to alter mRNA levels before single-cell capture, lysis and poly[T] priming. In addition, although communication between neighbouring cells can serve to maintain cellular states, scRNA-seq operates under the assumption that isolation of single cells away from such influences does not trigger rapid artefactual transcriptomic changes before mRNA capture. Thus, before embarking on a scRNA-seq study, researchers should aim to optimize the recovery of single cells from their target tissue, without excessive alteration to the transcriptome. It should also be noted that emerging studies have performed scRNA-seq on nuclei rather than intact single cells, which requires less tissue dissociation, and where nuclei were isolated in a manner that was less biased by cell type than single-cell dissociation [34, 35].

With regard to preserving single-cell transcriptomes before scRNA-seq, most published scRNA-seq studies progressed immediately from single-cell isolation to cell lysis and mRNA capture. This is clearly an important consideration for experimental design as it is not trivial to process multiple samples simultaneously from biological replicate animals or individual patients if labour-intensive single-cell isolation protocols such as FACS-sorting or micro-dissection are employed. Commercial droplet-based platforms might offer a partial solution as a small number of samples (for example, eight samples on the Chromium system) can be processed simultaneously. For samples derived from different individuals, SNP information might allow processing as pools, followed by haplotype-based deconvolution of cells [52]. Another possible solution might be to bank samples until such time as scRNA-seq processing can be conducted. To this end, recent studies have explored the effect of cryopreservation on scRNA-seq profiles and indeed suggest that high-fidelity scRNA-seq data can be recovered from stored cells [47, 53]. Furthermore, over the past few years, protocols compatible with certain cell-fixation methods have started to emerge [34, 35, 38, 54, 55].

## Which protocol should be employed?

As stated above, the nature of the research question plays an important role in determining which scRNA-seq protocol and platform should be employed. For example, prospective studies of poorly characterized heterogeneous tissues versus characterization of transcriptional responses within a specific cell population might be optimally served by different experimental approaches. Approximately 20 different scRNA-seq protocols have been published to date, the fine details of which have been thoroughly discussed elsewhere [56]. A key difference among these methods is that some provide full-length transcript data, whereas others specifically count only the 3’-ends of the transcripts (Table 1). Recent meta-analyses indicate that all of the widely used protocols are highly accurate at determining the relative abundance of mRNA transcripts within a pool [32, 33]. By contrast, significant variation was revealed in the sensitivity of each protocol. More specifically, the minimum number of mRNA molecules required for confident detection of gene expression varied between protocols, indicating that, for a given depth of sequencing (Box 1), some protocols are better than others at detecting weakly expressed genes [33]. In addition, certain transcripts that are expressed at low levels have been shown to be preferentially detected by using full-length transcript methods, potentially owing to having 3’-proximal sequence features that are difficult to align to the genome [32].

**Table 1:** Brief overview of scRNA-seq approaches

*Protocol example*	*C1* *(SMARTer)*	*Smart-seq2*	*MATQ-seq*	*MARS-seq*	*CEL-seq*	*Drop-seq*	*InDrop*	*Chromium*	*SEQ-well*	*SPLIT-seq*
*Transcript data*	Full length	Full length	Full length	3’-end counting	3’-end counting	3’-end counting	3’-end counting	3’-end counting	3’-end counting	3’-end counting
*Platform*	Microfluidics	Plate-based	Plate-based	Plate-based	Plate-based	Droplet	Droplet	Droplet	Nanowell array	Plate-based
*Throughput (number of cells)*	10^2^–10^3^	10^2^–10^3^	10^2^–10^3^	10^2^–10^3^	10^2^–10^3^	10^3^–10^4^	10^3^–10^4^	10^3^–10^4^	10^3^–10^4^	10^3^–10^5^
*Typical read depth* *(per cell)*	10^6^	10^6^	10^6^	10^4^–10^5^	10^4^–10^5^	10^4^–10^5^	10^4^–10^5^	10^4^–10^5^	10^4^–10^5^	10^4^
*Reaction volume*	Nanoliter	Microliter	Microliter	Microliter	Nanoliter	Nanoliter	Nanoliter	Nanoliter	Nanoliter	Microliter
*Reference*	[63]	[57]	[39]	[10]	[64]	[45]	[46]	[47]	[101]	[38]

Given that there are several scRNA-seq protocols, a few issues need to be considered in order to decide which one suits any particular researcher’s needs best. The first issue relates to the type of data that are required. Researchers interested in having the greatest amount of detail per cell should opt for protocols that are recognized for their high sensitivity, such as SMART-seq2 [32, 33, 57]. We emphasize, however, that almost all published scRNA-seq protocols have been excellent at determining the relative abundance of moderately to highly expressed transcripts within one cell. In some cases, including for splice-variant analysis, full-length transcript information is required, meaning that the 3’-end counting protocols would be discounted. In other applications, such as identification of cell types from complex tissues, maximising the throughput of cells is key. In such cases, the droplet-based methods hold an advantage, having relatively low cost per cell, which has an accompanying trade-off in reduced sensitivity.

A major issue common to all protocols is how to account for technical variation in the scRNA-seq process from cell to cell. Some protocols ‘spike-in’ (Box 1) a commercially available, well-characterized mix of polyadenylated mRNA species, such as External RNA Control Consortium (ERCC) controls (Ambion/Thermo Fisher Scientific) [58] or Spike-in RNA Variant Control Mixes (SIRVs, Lexogen). The data from spike-ins can be used for assessing the level of technical variability and for identifying genes with a high degree of biological variability [7]. In addition, spike-ins are valuable when computationally correcting for batch effects between samples [59]. However, the use of spike-ins is itself not without problems. First, one has to carefully calibrate the concentration that results in an optimal fraction of reads from the spike-ins. Second, spike-in mixes are sensitive to degradation, which can manifest as batch differences across temporally separated samples. Finally, spike-ins have been shown to be captured less efficiently than endogenous transcripts [33]. An increasingly popular method involves the use of UMIs, which effectively tags every mRNA species recovered from one cell with a unique barcode [41]. Theoretically, this allows estimation of absolute molecule counts, although the UMIs can be subject to saturation at high expression levels [33]. Nevertheless, the use of UMIs can significantly reduce amplification bias and therefore improve precision [32]. Both of these current techniques—spike-ins and UMIs—are generally accepted by the field, but it should be appreciated that they are not available for every protocol. In general, spike-in RNAs are not compatible with droplet-based approaches, whereas UMIs are typically used in protocols where only the 3’-ends of transcripts are sequenced, such as CEL-seq2, Drop-seq and MARS-seq [10, 45, 60].

## How many cells must I sequence and to what depth?

Two important questions that researchers face are ‘how many cells must I analyse?’ and the seemingly unrelated question ‘to what depth must my sequencing analysis be performed?’ The answers to these questions are in fact intertwined. Given that most scRNA-seq data are generated by sequencing cDNA libraries from single cells that are barcoded and pooled, the depth of single-cell sequencing (that is, the number of transcripts detected from each cell) diminishes as the number of libraries included in a sequencing run is increased, owing to a finite sequencing capacity per run.

As a rule of thumb, the required number of cells increases with the complexity of the sample under investigation. In a heterogeneous population of cells, for example T lymphocytes that express highly diverse antigen receptors, it might be difficult to observe relationships between transcriptomes, and, in such instances, a larger number of cells will provide greater statistical power and opportunity to observe patterns. In some cases, heterogeneity can be reduced by experimental design. For example, in recent studies of murine T-cell responses in vivo, this issue was circumvented by employing transgenic T-cell receptor cells that expressed the same antigen receptor [24, 61]. Clearly, it can be difficult to predict the degree of heterogeneity that will be revealed by a scRNA-seq study. However, it might be possible, for example, to perform power calculations and group size estimates if other single-cell data, such as flow- or mass-cytometric data, are available [62].

While the required number of cells is dependent on the number of distinct cell states within the population, the required sequencing depth also depends on the magnitude of differences between these states. For example, unbiased cell-type classification within a mixed population of distinct cell types can be achieved with as few as 10,000 to 50,000 reads per cell [10, 63]. Indeed, increasing the cell numbers to be assessed, yet keeping the read depth relatively low, provides increasing power at detecting populations that exist at a frequency of < 1% of the total population. Therefore, opting for a lower read depth is practical and economical if the goal of the study is to identify rare cell populations or to scan cells for evidence of mixed populations. However, lower read depths will not necessarily provide detailed information on gene expression within any given single cell, and many biological processes associated with more-subtle transcriptional signatures necessitate deeper sequencing. It is at this point that the ‘*zero* or *dropout* problem’ (Box 1) of scRNA-seq should be raised. The efficiency with which poly-adenylated mRNA species are captured, converted into cDNA and amplified is currently unclear, and, depending on the study, can range between 10 and 40% [13, 44, 64, 65]. This means that, even if a gene is being expressed, perhaps at a low level, there is a certain probability that it will not be detected by current scRNA-seq methods. A partial solution to this issue is to increase read depth. However, beyond a certain point, this strategy leads to diminishing returns as the fraction of PCR duplicates increases with deeper sequencing. Current data suggest that single-cell libraries from all common protocols are very close to saturation when sequenced to a depth of 1,000,000 reads, and a large majority of genes are detected already with 500,000 reads, although the exact relationships are protocol specific [32, 44].

However, the confidence in whether a gene is truly expressed, or not, depends on how many mRNA molecules are detectable, which is dependent on many factors, including mRNA stability. The data suggest that, if the main goal of the study is to characterize the transcriptome of a particular cell with the greatest possible resolution, then a median read depth of around one million is essential. It should be noted that researchers can also employ lower read-depth datasets to explore on a population level whether a given gene appears to be expressed within cell populations. Thus, gene-specific information can be extracted from lower read-depth datasets. However, more-detailed examination of gene–gene co-expression and co-regulation or differential gene splicing requires high read depths.

To date, most scRNA-seq studies employing higher read depths examined hundreds to thousands of cells, for reasons of cost and platform availability. Increasingly, lower read-depth-based studies are emerging that examine 10–100-fold more cells [10, 45–47], particularly with droplet-based technologies. Researchers should consider which of these ranges best suits their biological system, their questions and their budget.

## How does single-cell data differ from bulk RNA-seq?

While scRNA-seq workflows are conceptually closely related to population-level transcriptomics protocols, data from scRNA-seq experiments have several features that require specific bioinformatics approaches. First, even with the most sensitive platforms, the data are relatively sparse owing to a high frequency of dropout events (lack of detection of specific transcripts). Moreover, owing to the digital nature of gene expression at the single-cell level, and the related phenomenon of transcriptional bursting (in which pulses of transcriptional activity are followed by inactive refractory periods; Box 1), transcript levels are subject to temporal fluctuation, further contributing to the high frequency of zero observations in scRNA-seq data. Therefore, the numbers of expressed genes detected from single cells are typically lower compared with population-level ensemble measurements. Because of this imperfect coverage, the commonly used unit of normalized transcript levels used for bulk RNA-seq, expressed as ‘reads per kilobase per million’ (RPKM), is biased on a single-cell level, and instead the related unit ‘transcripts per million’ (TPM) should be used for scRNA-seq [66].

Second, scRNA-seq data, in general, are much more variable than bulk data. scRNA-seq data typically include a higher level of technical noise (such as dropout events), but also reveal much of the biological variability that is missed by RNA-seq on pooled cells. Biological variation is present on many levels, and which of these are considered as nuisance variation depends on the underlying biological question being asked. For example, at the gene level, transcriptional bursting causes variation in transcript quantities [67], whereas at the global level, the physical size of individual cells can vary substantially, affecting absolute transcript numbers and reflected in the number of detected genes per cell [68, 69]. Cell-size variation can also be closely related to proliferative status and cell-cycle phase. Several computational approaches have been devised that account for such variability [59, 70, 71]. Typically, the most biologically interesting heterogeneity among cells, other than heterogeneity in lineage identity, is due to different intermediate transcriptional states, which can provide information about whether the regulation of individual cells is normal or aberrant. Although the distinction between these states can in some cases be blurred, in general these are associated with subtle transcriptional changes that warrant greater sequencing depth for their resolution [72].

Finally, distributions of transcript quantities are often more complex in single-cell datasets than in bulk RNA-seq. In general, single-cell expression measurements follow a negative binomial distribution [73], and, in heterogeneous populations, multimodal distributions are also observed [74]. As a consequence, statistical tests that assume normally distributed data (used for example for detecting differentially expressed genes) are likely to perform suboptimally on scRNA-seq data.

## Once I have sequenced my single-cell cDNA libraries, how do I analyse the data?

Although scRNA-seq is now more accessible to ‘first-time’ researchers through commercial reagents and platforms, this is less true for the crucial bio-informatic and computational demands of a scRNA-seq study. There are currently very few, if any, ‘plug-and-play’ packages that allow researchers to quality control (QC), analyse and interpret scRNA-seq data, although companies that sell the wet-lab hardware and reagents for scRNA-seq are increasingly offering free software (for example, Loupe from 10x Genomics, and Singular from Fluidigm). These are user-friendly but have the drawback that they are to some extent a ‘black box’, with little transparency as to the precise algorithmic details and parameters employed. Nevertheless, this is a highly dynamic area, where gold-standard analysis platforms are yet to emerge. Recent reports indicate that more-user-friendly, web-browser-based interfaces will become available soon [75]. However, the precise functionalities that need to be offered continue to be an area of active development. In summary, an understanding of the bioinformatic and computational issues involved in scRNA-seq studies is needed, and specialist support for biomedical researchers and clinicians from bio-informaticians who are comfortable with handling scRNA-seq datasets would be beneficial.

Before further analyses, scRNA-seq data typically require a number of bio-informatic QC checks, where poor-quality data from single cells (arising as a result of many possible reasons, including poor cell viability at the time of lysis, poor mRNA recovery and low efficiency of cDNA production) can be justifiably excluded from subsequent analysis. Currently, there is no consensus on exact filtering strategies, but most widely used criteria include relative library size, number of detected genes and fraction of reads mapping to mitochondria-encoded genes or synthetic spike-in RNAs [76, 77]. Recently, sophisticated computational tools for identifying low-quality cells have also been introduced [78–81]. Other considerations are whether single cells have actually been isolated or whether indeed two or more cells have been mistakenly assessed in a particular sample. This can sometimes be assessed at the time of single-cell isolation, but, depending on the chosen technique, this might not always be possible.

Once the scRNA-seq data are filtered for poor samples, they can be interpreted by an ever-increasing range of bio-informatic and computational methods, which have been reviewed extensively elsewhere [74, 82]. The crux of the issue is how to examine tens of thousands of genes possibly being expressed in one cell, and provide a meaningful comparison to another cell expressing the same large number of genes, but in a very different manner. Most approaches seek to reduce these ‘multi-dimensional’ data, with each dimension being the expression of one gene, into a very small number of dimensions that can be more easily visualised and interpreted. Principal component analysis (PCA) is a mathematical algorithm that reduces the dimensionality of data, and is a basic and very useful tool for examining heterogeneity in scRNA-seq data. This has been augmented by a number of methods involving different machine-learning algorithms, including for example t-distributed stochastic neighbour embedding (t-SNE) and Gaussian process latent variable modelling (GPLVM), which have been reviewed in detail elsewhere [74, 82, 83].

Dimensionality reduction and visualization are, in many cases, followed by clustering of cells into subpopulations that represent biologically meaningful trends in the data, such as functional similarity or developmental relationship. Owing to the high dimensionality of scRNA-seq data, clustering often requires special consideration [84], and a number of bespoke methods have been developed [45, 85–88]. Likewise, a variety of methods exist for identifying differentially expressed genes across cell populations [89].

An increasing number of algorithms and computational approaches are being published to help researchers define the molecular relationships between single cells characterized by scRNA-seq and thus extend the insights gained by simple clustering. These trajectory-inference methods are conceptually based on identification of intermediate cell states, and the most recent tools are able to trace both linear differentiation processes as well as multipronged fate decisions [22, 24, 90–95]. While these approaches currently require at least elementary programming skills, the source codes for these methods are usually freely available for bio-informaticians to download and use. This reinforces the need to cultivate a good working relationship with bio-informaticians if scRNA-seq data are to be analysed effectively.

## What will the next 5 years hold for scRNA-seq?

Over the past 6 or so years, there has been an explosion of interest in using scRNA-seq to provide answers to biologically and medically related questions, both in experimental animals and in humans. Many of the studies from this period either pioneered new wet-lab scRNA-seq protocols and methodologies or reported novel bio-informatic and computational approaches for quality-controlling and interpreting these unique datasets. Some studies also provided tantalizing glimpses of new biological phenomena that could not have been easily observed without scRNA-seq. Here, we consider what the next 5 years might hold for scRNA-seq from the perspective of clinical and experimental researchers looking to use this technology for the first time.

Given that the field of single-cell genomics is experiencing rapid growth, aside from being confident that numerous advances will be made, exactly what these will be remains difficult to predict. Nevertheless, we point towards various areas in which we hope and expect numerous advances to be made. First, most scRNA-seq studies have tended to examine freshly isolated cells. We expect many more studies will explore cryopreserved and fixed tissue samples using scRNA-seq, which will further open up this technology to clinical studies.

As isolation of single cells is of paramount importance to this approach, we expect more advances in wet-lab procedures that rapidly dissociate tissue into individual cells without perturbing their transcriptomes. In addition, while many scRNA-seq studies have employed expensive hardware, including microfluidic and droplet-based platforms, future studies will reduce costs by further reducing reaction volumes, and perhaps also by avoiding the need for bespoke pieces of equipment [38]. Currently, much of the cost associated with conducting a scRNA-seq study is associated with cDNA library preparation and NGS. Given ongoing trends for decreasing sequencing costs, we anticipate that these cost benefits will also make scRNA-seq more affordable on a per-cell basis. This will likely drive another trend—the ever-increasing number of cells examined in a given study. While early studies examined a few hundred cells, with reduced costs and the widespread adoption of newer droplet-based technologies, we anticipate that analysis of millions to billions of cells will become commonplace within the next 5 years [96]. The Human Cell Atlas project [51], with the ultimate goal of profiling all human cell states and types, is evidence of this trend. With the accumulation of such enormous datasets, the issue arises regarding how to use them to their full potential. Many researchers would without doubt benefit from centralized repositories where data could be easily accessed at the cellular level instead of just sequence level [97].

Next, as mentioned above, the ‘drop-out’ problem that occurs even in high-resolution scRNA-seq datasets illustrates that weakly or even moderately expressed genes can be missed, partly owing to the currently modest efficiencies for mRNA capture. We expect that mRNA capture rates will continue to improve over the next 5 years, to an extent where perhaps almost all mRNA molecules will be captured and detected. This will permit more-sensitive analysis of gene expression in individual cells and might also serve to reduce the number of cells required in any given study.

Given the unique analytical challenges posed by scRNA-seq datasets, we expect great advances in bioinformatic and computational approaches in the coming years. In particular, user-friendly, web-browser-like interfaces will emerge as gold-standard packages for dealing with scRNA-seq data. These will contain all the necessary functionality to allow researchers first to QC their data and then to extract biological information relating to heterogeneity, the existence of rare populations, lineage tracing, gene–gene co-regulation and other parameters.

Recent studies are providing exciting possibilities for combining scRNA-seq with other modalities. For instance, the use of CRISPR–Cas9 genome-editing techniques alongside barcoded guide RNA species has allowed high-throughput assessment of gene function in single cells [98, 99]. We expect that many new combination approaches will emerge using proteomics, epigenomics and analysis of non-coding RNA species alongside scRNA-seq (reviewed in [100]). We speculate that the next decade will take us closer to a truly holistic examination of single cells, which takes into account not only mRNA, but also the genome, epigenome, proteome and metabolome.

Finally, we believe that several clinical applications will emerge for scRNA-seq in the next 5 or so years. For example, resected tumours might be routinely assessed for the presence of rare malignant and chemo-resistant cancer cells. This information will provide crucial diagnostic information and will guide decisions regarding treatment. Next, as an extension to a full blood count, scRNA-seq assessments will provide in-depth information on the response of immune cells, which again will inform diagnoses and the choice of therapy. Finally, the relatively small numbers of cells present in a range of other tissue biopsies, for example from the skin and gut mucosal surfaces, will be ideal for providing molecular data that informs on diagnosis, disease progression and appropriate treatments. Thus, scRNA-seq will progress out of specialist research laboratories and will become an established tool for both basic scientists and clinicians alike.

## Conclusions

This decade has marked tremendous maturation of the field of single-cell transcriptomics. This has spurred the launch of numerous easily accessible commercial solutions, increasingly being accompanied by dedicated bioinformatics data-analysis suites. With the recent advances in microfluidics and cellular barcoding, the throughput of scRNA-seq experiments has also increased substantially. At the same time, protocols compatible with fixation and freezing have started to emerge. These developments have made scRNA-seq much better suited for biomedical research and for clinical applications. For example, the ability to study thousands of cells in a single run has greatly facilitated prospective studies of highly heterogeneous clinical samples. This can be expected to have a profound impact on both translational applications as well as our understanding of basic tissue architecture and physiology. With these increasing opportunities for single-cell transcriptome characterization, we have witnessed remarkable diversification of experimental protocols, each coming with characteristic strengths and weaknesses. Researchers therefore face decisions such as whether to prioritize cell throughput or sequencing depth, whether full-length transcript information is required, and whether protein-level or epigenomic measurements are to be performed from the same cells. Having clearly defined biological objectives and a rational experimental design are often vital for making an informed decision about the optimal approach.

## Abbreviations

mRNA: Messenger RNA
NGS: Next-generation sequencing
QC: Quality control
RNA-seq: RNA sequencing
scRNA-seq: Single-cell RNA sequencing
SNP: Single-nucleotide polymorphism
UMI: Unique molecular identifier
